# Hospital Worthies

**Published:** 1887-05-28

**Authors:** 


### Hospital Worthies.

### MESSENGER MONSEY, M.D., F.R.S.

**Introductory.** In our notices of "Hospital Worthies" it is not our intention to charge our pages with profitless biographical *data*, though a few brief facts may perhaps be desirable, our main purpose being to deal succinctly with interesting episodes and reminiscences in the lives of doctors of the present and of by-gone times.

**A Fortunate Start.** Messenger Monsey was the son of the Rev. Robert Monsey, a non-juring clergyman, and was born in the year 1693. His father appears to have been a plain, homely man, imbued with the belief that his boy was a genius who was destined to take the world by storm. The lad, after receiving a sound classical education under the parental eye, was sent to Pembroke College, Cambridge, where he took his B.A. degree in 1714. On leaving the University he returned to his native county, Norfolk, and studied physic for some time under Sir Benjamin Wrench, and afterwards opened practice for himself at Bury St. Edmunds. Whilst there a strange and very fortunate circumstance occurred, a circumstance which launched the Doctor on the great highway to success. Lord Godolphin, a grandson of the great Duke of Marlborough, chanced, whilst journeying to his seat at Gogmagog, near Newmarket, to be seized with an attack of apoplexy. Bury was the nearest town, and to Bury accordingly his lordship's messengers went in search of a disciple of Celsus, and here it was they found the then unknown Dr. Messenger Monsey. The Doctor's treatment was exceedingly successful. During his recovery his lordship entered into conversation with his physician, whom he was surprised to find a man of distinguished parts. His lordship's friendship with the Doctor was as rapid as his convalescence. Godolphin insisted that Monsey should come to London, promising him his patronage and influence. Just about this time a vacancy occurred at Chelsea Hospital, and this was accordingly filled by the nomination of Godolphin's bosom friend.

**Fifty Years' Office.** For the lengthened period of half a century Dr. Messenger Monsey presided over this great establishment. His fame spread from the little village on the banks of the Thames over the great town. Sir Robert Walpole, the Earl of Chesterfield, Dr. Samuel Johnson, and many other great men of the age, hastened to make the acquaintance of the man whose talents and eccentric mannerisms everyone talked of.

Walpole appears to have appreciated his worth. The two were great friends, often dining and playing at billiards together. The Doctor had an unpleasant knack of contradicting, which at times somewhat irritated the Prime Minister. Monsey was a very clever billiard player, and in his contests with Sir Robert the latter generally came off second best. "How happens it," remarked Walpole one day in Monsey's hearing, "that nobody contradicts me or beats me at billiards but Dr. Monsey?" "They get places," rejoined the Doctor, "while I get a dinner and praise." When he liked, Dr. Monsey could be one of the most agreeable and affable of men, brimful of humour and anecdote, and possessing an inexhaustible flow of language and a memory of extraordinary retentiveness. At other times his wit chided with offensiveness. Monsey was no respecter of persons, and when he punished it was with a rod of scorpions.

**The Doctor's Eccentricity.** Eccentricity of character, in a more or less pronounced form, is far more common than the ordinary observer may suppose. In foremost natures there is a certain deviation from that which is regular and stated. Sometimes the departure from the normal standard is spasmodic and occasional only; at other times it dominates and directs the whole course of a man's life.

In the case of Dr. Messenger Monsey we find perhaps the strangest combination of talent and eccentricity which the medical annals of this or any other country can produce. In his day—and it was a very long day, too—the "Norfolk Doctor" was the talk of the town; now, however, his memory has well-nigh perished.

There are endless anecdotes told of this old hospital worthy. One of the most curious is with regard to the mode he adopted of drawing his own teeth. He would attach one end of a piece of catgut around the decayed molar, while the other was fixed to a perforated bullet. With this he would load his pistol and fire. One day he persuaded a friend to allow him to extract an aching tooth by this novel process. As he was posing the pistol, the patient exclaimed, "Hold! stop! I have altered my mind." "But I have not," said Monsey, and away went the offending bicuspid. The Doctor took delight in telling a story of the humble origin of his ancestors, one of whom combined the trades of baker and hop-factor. So straitened became the circumstances of this dual trader, that he was once forced, when the hop-market was flat, to sell the feathers out of his beds, and fill the ticks with his unsold hops. Some time afterwards there was a blight among the hops, which rose to a fabulous price in the market. The Doctor's ancestor turned the hops out of his beds, and sold them for a good round sum. "And thus," Dr. Monsey would close his story, "our family *hopped* from obscurity."

Dr. Monsey held, as we have said, the physicianship of Chelsea Hospital for fifty years. The reversion of his office was promised from time to time to many place-hunters, but the old Doctor continued to outlive them all. He believed that he should live to be a hundred, and he nearly attained his wish. Looking out from one of the windows of Chelsea College one morning, he saw a gentleman taking a view of his "land of promise," and examining carefully the grounds and premises. Surmising the circumstances, the Doctor hurried out to disabuse the mind of the scrutiniser of any such vain hopes.

The Reversion of the Situation.

"Well, sir," said Monsey, "I see you are examining your house and gardens that *are to be*, and I can assure you that they are both very pleasant and very convenient. But I must tell you one circumstance: you are the fifth man that has had the reversion of the place. I have buried them all,—and what is more," he continued, carefully regarding him, "there is something in your face which tells me that I shall bury you too." On another occasion the Doctor thus accosted a gentleman who had gone down to inspect the situation: "So, sir, I find you are one of the candidates to succeed me." The physician bowed assent. "But you will be confoundedly disappointed." "Disappointed?" reiterated the physician, with quivering lips. "Yes," rejoined the old Doctor. "You expect to outlive me, and I can discern in your countenance, and from other concomitant circumstances, that you are deceiving yourself. You will certainly die first,—though I have nothing to expect from that event. I shall not rejoice at your death, as I am persuaded you would at mine." Both of the expectant physicians died before Monsey; and when his own death actually occurred, so much silly superstition had gathered round the reversion, that there was no one in waiting for his shoes.

Dr. Monsey was somewhat of a miser, and his eccentric habits led him to conceal his wealth in all kinds of out-of-the-way places. One summer, when he journeyed to his paternal home, he determined, so runs the story, to hide a roll of bank-notes in his sitting-room grate,—for at that period of the year he felt that there was no possibility of the fire being lit. The old woman who had charge of his rooms chanced to have a friend in, and being desirous of treating her with more than ordinary respect, determined to boil a kettle of water for tea in her master's sitting-room. Monsey returned home unexpectedly, and just in the nick of time. He rushed to the pump, and a pail of water soon extinguished the bundle of sticks, which were beginning to burn in the grate. The notes were safe, though slightly damaged.

Dr. Monsey died at the age of ninety-five, on the 26th December 1788, retaining all his faculties and all his whimsicalities to the last. His death was sudden. On the morning of the day, he observed to the maid who brought his breakfast, "I shall certainly lose the game." The girl inquired what he meant. "The game of *a hundred*," replied Monsey,—"a game which I have played for very earnestly many years, but I shall lose it now, for I expect to die in a few hours."

Exit the Doctor.

